# Soluble adenylyl cyclase: A novel player in cardiac hypertrophy induced by isoprenaline or pressure overload

**DOI:** 10.1371/journal.pone.0192322

**Published:** 2018-02-21

**Authors:** Ilona Schirmer, Tippaporn Bualeong, Heidi Budde, Diana Cimiotti, Avinash Appukuttan, Nicole Klein, Philip Steinwascher, Peter Reusch, Andreas Mügge, Rainer Meyer, Yury Ladilov, Kornelia Jaquet

**Affiliations:** 1 Cardiology, Research Laboratory Molecular Cardiology, BG Bergmannsheil and St. Josef-Hospital, clinics of the Ruhr-University Bochum, Bochum, Germany; 2 Institute of Physiology II, Hospital of the Friedrich-Wilhelms-University of Bonn, Bonn, Germany; 3 Clinical Pharmacology, Faculty of Medicine, Ruhr-University of Bochum, Bochum, Germany; Maastricht University, NETHERLANDS

## Abstract

**Aims:**

In contrast to the membrane bound adenylyl cyclases, the soluble adenylyl cyclase (sAC) is activated by bicarbonate and divalent ions including calcium. sAC is located in the cytosol, nuclei and mitochondria of several tissues including cardiac muscle. However, its role in cardiac pathology is poorly understood. Here we investigate whether sAC is involved in hypertrophic growth using two different model systems.

**Methods and results:**

In isolated adult rat cardiomyocytes hypertrophy was induced by 24 h β_1_-adrenoceptor stimulation using isoprenaline (ISO) and a β_2_-adrenoceptor antagonist (ICI118,551). To monitor hypertrophy cell size along with RNA/DNA- and protein/DNA ratios as well as the expression level of α-skeletal actin were analyzed. sAC activity was suppressed either by treatment with its specific inhibitor KH7 or by knockdown. Both pharmacological inhibition and knockdown blunted hypertrophic growth and reduced expression levels of α-skeletal actin in ISO/ICI treated rat cardiomyocytes. To analyze the underlying cellular mechanism expression levels of phosphorylated CREB, B-Raf and Erk1/2 were examined by western blot. The results suggest the involvement of B-Raf, but not of Erk or CREB in the pro-hypertrophic action of sAC. In wild type and sAC knockout mice pressure overload was induced by transverse aortic constriction. Hemodynamics, heart weight and the expression level of the atrial natriuretic peptide were analyzed. In accordance, transverse aortic constriction failed to induce hypertrophy in sAC knockout mice. Mechanistic analysis revealed a potential role of Erk1/2 in TAC-induced hypertrophy.

**Conclusion:**

Soluble adenylyl cyclase might be a new pivotal player in the cardiac hypertrophic response either to long-term β_1_-adrenoceptor stimulation or to pressure overload.

## Introduction

Cyclic adenosine monophosphate (cAMP) signaling plays an essential role in proliferative and non-proliferative cell growth, and is involved in the development of cardiac hypertrophy in the cardiovascular system [1,2].

Two classes of cyclases synthesize cAMP in mammalian cells, the transmembrane adenylyl cyclase (tmAC) and the soluble adenylyl cyclase (sAC). In contrast to tmAC, sAC does not possess a transmembrane domain [3], and is insensitive to the response of heterotrimeric G- proteins to hormonal stimuli or forskolin treatment [4]; however, it senses intracellular levels of bicarbonate and ATP [5,6]. Furthermore, sAC can be activated by calcium (Ca^2+^) and manganese ions (Mn^2+^) [7,8]. Recently, the structure of the catalytic domain was solved [9]. Its overall structure is similar to adenylyl cyclases in cyanobacteria, but not to mammalian tmACs, and several splicing isoforms exist [3,10]. Full-length sAC (ca. 180 kDa) is predominant in testis, whereas the main truncated isoform consisting essentially of the two catalytic domains (ca. 50 kDa) is present in most other tissues [11,12].

tmACs produce cAMP exclusively upon an extracellular signal. In contrast, sAC, which is localized in different intracellular compartments (e.g. cytosol, mitochondria, and nucleus) [13], enables cAMP production in cell compartments distant to the plasmalemma independent of extracellular signals, and as such, might be involved in various signaling pathways. Ever since sAC has been isolated from the cytosolic fractions of testis [14,15], its function has been investigated in numerous tissues and cells [16–19]. But its physiological role in cardiac muscle remains largely unknown. Initial studies revealed a role for sAC in the regulation of the heart rate in the pacific hagfish [20], in anoxia/re-oxygenation-induced apoptosis of cardiomyocytes [21] and coronary endothelial cells [22]. Aside from cell death, sAC controls axonal growth in neurons and prostate epithelial cell proliferation [23]. Importantly, in prostate cells, sAC promotes proliferation through activation of exchange protein activated by cAMP (Epac)/rapidly accelerated fibrosarcoma (B-Raf)/extracellular-signal regulated kinase (Erk) signaling [19, 24]. Given that the Erk pathway participates in isoprenaline (ISO)-induced cardiac hypertrophy in neonatal cardiomyocytes [1], it can be presumed that in terminally differentiated cardiomyocytes, sAC-dependent activation of B-Raf/Erk signaling may contribute to hypertrophic growth. Furthermore, Zippin et al. [25] demonstrated that sAC controls the activity of the cAMP response element binding protein (CREB) via protein kinase A (PKA)-dependent phosphorylation in the nucleus of COS7 cells. The fact that CREB is also involved in the hypertrophic growth of cardiomyocytes [26], is a further clue for a possible role of sAC in cardiac hypertrophy. Therefore, in the present study, we investigated if sAC plays a role in cardiac hypertrophy. We used two completely different hypertrophy models, namely an *in vitro* model (isolated adult rat cardiomyocytes) with ISO/ICI induced hypertrophy and an *in vivo* model (mice) with hypertrophy induced by pressure overload. Based on our results, we suggest that sAC is pivotally involved in the development of hypertrophy in the *in vitro* and the *in vivo* model.

## Methods

Treatment of rats to isolate cardiomyocytes as well as handling and housing of mice for TAC experiments were performed in consent with local authorities of Ruhr-University of Bochum and Friedrich-Wilhelms-University of Bonn, respectively, and to the directive of the EU (2010/63/EU) and NIH (guide of care and use of laboratory animals, 8th ed, 2011). Rats were anesthetized by isoflurane (2 vol%) before decapitation to isolate cardiomyocytes; Reference number for mice experiments (TAC, performed in Bonn): 87–51.04.A048.

If not mentioned otherwise, all reagents were obtained from Sigma-Aldrich (Hamburg, Germany).

### Isolation of adult rat cardiomyocytes

12 weeks old male Wistar Kyoto rats (Charles River (Germany)) were anaesthetized using isoflurane (2 vol%), and then were sacrificed by decapitation to isolate cardiac myocytes from ventricles as previously described [27].

### Induction of hypertrophy and pharmacological inhibition of sAC

To induce hypertrophy, cells were treated for 24 h with the β-adrenoceptor agonist isoprenaline (ISO, 10 μmol/L) and the β_2_-adrenoceptor antagonist ICI 118,551 (ICI, 0.05 μmol/L) [28]. For specific inhibition of sAC activity, cells were treated with KH7 (12.5 μmol/L; Cayman Chemicals, Ann Arbor, Michigan, USA). This concentration has been chosen as it reduced sAC activity by > 50% but does not affect tmACs [29]. Long-term treatment with >15 μmol/L KH7 had a cytotoxic effect, which might be due to suppression of mitochondrial function [30].

### sAC knockdown

To express sAC-targeted small hairpin RNA (shRNA) and scrambled shRNA (scRNA) in isolated adult rat cardiomyocytes, adenoviral vectors were constructed using the AdEasy Adenoviral Vector System according to He et al. [31] as previously described [21,27]. The recombinant viruses were propagated in HEK293 cells and recovered after repeated freeze–thaw cycles. The required volume of virus stock was checked by analysis of transfection efficiency as described formerly [27]. Cardiomyocytes were infected using 100 μL of the viral stock per mL culture medium to overexpress either shRNA or scRNA. 72 h after infection ISO/ICI were added.

### Analysis of hypertrophy

To verify hypertrophic growth, protein/DNA and RNA/DNA ratios, cross sectional cell area, and the expression level of α-skeletal muscle actin as a marker for hypertrophy were determined. The cross-sectional cell area was analyzed using an Axiovert 100M microscope connected to a camera with the appropriate software (Axio vision Rel. 4.8; Zeiss, Göttingen, Germany). 35–40 representative cells/dish were analyzed. The peqGold TriFast Fl (Peqlab, Erlangen, Germany) was used to purify RNA, DNA, and protein from cardiomyocytes (cells from a dish containing 500,000 cells were lysed). Additionally, protein and DNA was isolated from cardiomyocytes using the MasterPure Complete kit (Epicentre, Germany) for analysis of hypertrophy dependent on KH7 concentration. The concentration (ng/μL) and quality of isolated RNA and DNA were determined using the NanoDrop 1000 (Peqlab, Erlangen, Germany), and the protein concentration (μg/μL) was determined with the BCA Protein-Assay Kit (Thermo Fisher, Schwerte, Germany

### Western blot analysis

If not mentioned otherwise antibodies were purchased from Cell Signaling; Danvers, USA).

For western blotting, cells were lysed in a buffer containing 65.5 mmol/L Tris, 2% SDS, 10% glycerol, 50 mmol/L DTT, and 0.01% bromophenol blue followed by sonication (3 × 40 s). Lysates were incubated for 5 min at 95°C, and pelleted at 4°C for 15 min at 20,000 × g. Before blotting, 25 μg total protein was resolved on a SDS-PAGE gel. Equivalent sample loading was confirmed either by evaluation of reference band intensity using anti-GAPDH (Promega, Mannheim, Germany; 1:10^3^ dilution), anti-actin, or anti-tubulin antibody (1:10^3^ dilution; Millipore, Schwalbach, Germany) after removing antibodies from the membranes with stripping buffer (Pierce, Rockford, USA) or by evaluation of protein band intensities/lane, i.e., total proteins, via gel staining with Mini-PROTEAN^®^ TGX Stain-Free^™^ assay (BioRad, Germany). The primary antibodies used were: anti-sAC (clone K2 designed at the Ruhr-University of Bochum, clone R21(CEP Biotech) or clone R40, kindly provided by Dr. Lonny Levin and Dr. J. Buck, Cornell University, NY), anti-α-skeletal actin (Millipore; 1:10^3^ dilution), anti-atrial natriuretic peptide (ANP; Millipore;), anti-phospho-CREB (pCREB; 1:10^3^ dilution), anti-phospho-B-Raf (Ser445;pB-Raf), anti-B-Raf (St. Johns Laboratories, Great Britain, dilution of 1:500), anti-phospho-Erk1/2 (TEY-motif; P-Erk1/2), anti-Erk1/2 (1:500 dilution). Bands were visualized by infrared fluorescence (Odyssey; LI-COR Bioscience, Lincoln, USA) or by chemiluminescence (ECL Plus kit, Thermo Fisher).

### Transverse aortic constriction

Wild type (WT) and sAC knockout (KO) mice (genetic background 129SvEvBrd; strain: Adcy10^TM1Lex^) were purchased from Texas Institute for Genomic Medicine (Huston, USA). The mice were housed in polycarbonate transparent pathogen-free cages (365 × 207 × 140 mm) with animal bedding (ASBE-wood GmbH, Ahrensfelde, Germany) at a room temperature of 20–22°C and 50% humidity, and were maintained on a 12-hour day–night cycle. Mice were given free access to standard rodent chow and water *ad libitum*.

For transverse aortic constriction (TAC), mice were anaesthetised with isoflurane (2 vol%). To decrease the aortic diameter, a thread was wrapped around the aortic arch. A 27-gauge needle was used to standardise the degree of aortic constriction [32]. For analgesia, buprenorphine was administered by intraperitoneal injection (0.065 mg/kg). Sham control animals underwent surgery without aortic restriction. The mean age of mice at the time of surgery did not differ significantly between the groups and was (in weeks): WT sham: 14.1 ± 0.4 (n = 9); WT TAC: 13.9 ± 0.4 (n = 12); KO sham: 13.7 ± 0.7 (n = 9); KO TAC: 13.2 ± 0.3 (n = 9). WT and KO male mice were subjected to TAC for 2 weeks to induce cardiac hypertrophy. The mean body weights did not differ significantly between the groups and were as follows in g: WT sham: 28.7±0.64 (n = 9); WT TAC: 27.2 ±0.5 (n = 12); KO sham: 30.7 ± 1.20 (n = 9); KO TAC: 30.4 ±1.1 (n = 9).

Then, heart rate (HR), systolic arterial pressure (SAP), diastolic arterial pressure (DAP), left ventricular systolic pressure (LVSP), and left ventricular end-diastolic pressure (LVEDP) were recorded using an Ultra-Miniature Pressure Catheter (Scisense advancing micro-sensor technology^™^, USA/CAN) under mild anaesthesia (1% isoflurane, heart rate: ~500 min^–1^). Morphometric parameters such as body weight (BW), wet heart weight (HW), wet lung weight (LW) and tibia length (TL) were evaluated immediately upon excision of heart and lung under anesthetisation with isoflurane (2.5 vol%).

For western blot analyses hearts were snap frozen and kept at -80°C. Cardiac tissue lysates at 2 weeks after sham operation or transverse constriction (TAC) were examined for expression of ANP and phosphorylated Erk. For the left ventricular wall thickness analysis, the excised hearts were fixed in EGTA-Tyrode’s solution supplemented with 4% paraformaldehyde for 24 hours followed by dehydration in a graded series of ethanol (30%, 50%, and 70%) for 24 hours each step. Finally the hearts were embedded in paraffin, and 5 μm-thick sections were cut. Masson’s trichrome staining was performed to evaluate the thickness of the left ventricle wall.

### Statistics

Statistical analysis was performed using one way ANOVA with Newman-Keuls or Dunnett post-hoc test, the unpaired, two-tailed Student’s t-test applying GraphPad Prism software (Graph Pad Inc, La Jolla, USA) or) or a two way Anova (SigmaPlot, version 11). Data are expressed as means ± standard error of the mean (SEM).

## Results

### Effect of sAC inhibition on hypertrophic growth of isolated adult rat cardiomyocytes

**Fig 1:**
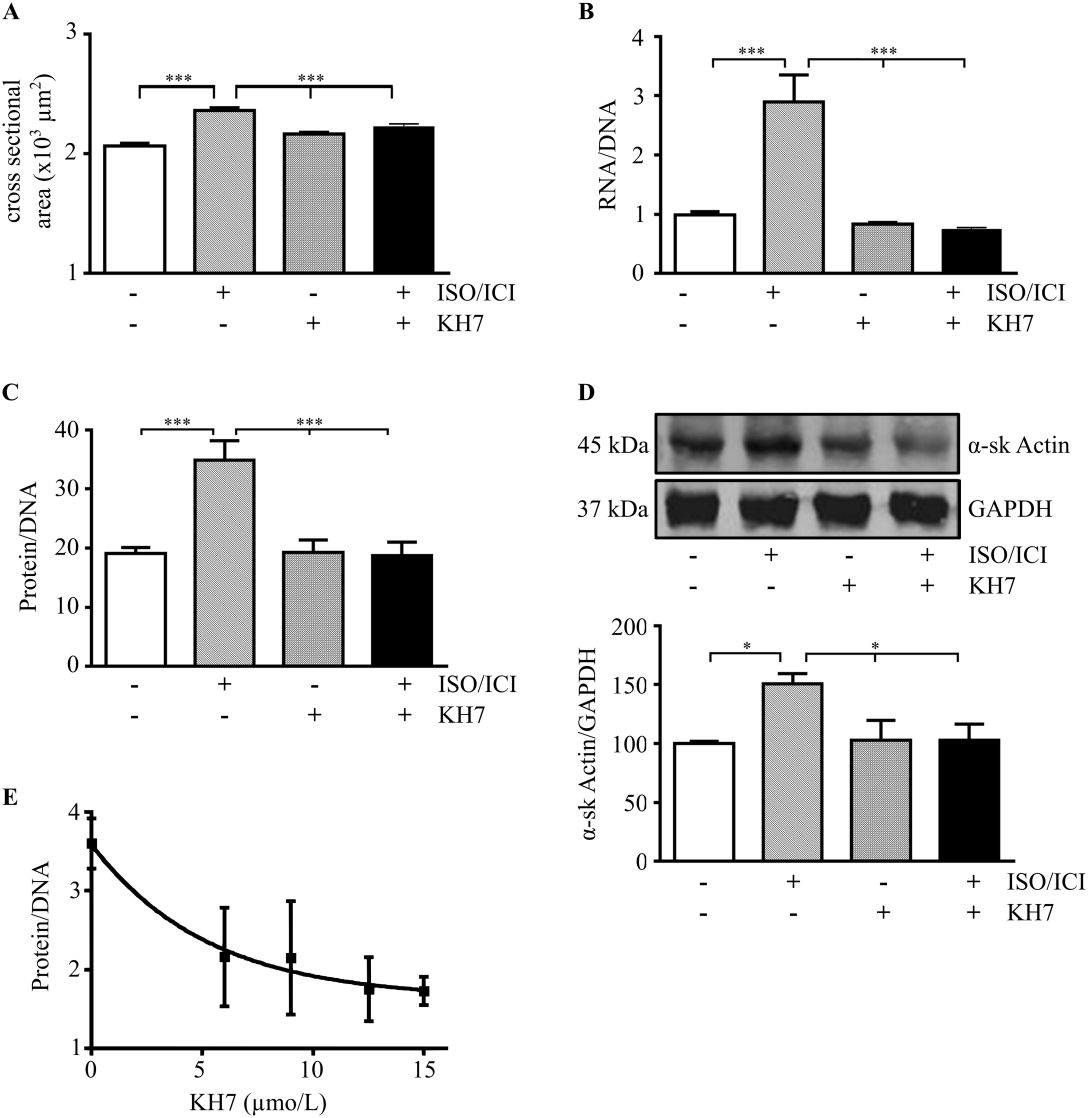
Effect of pharmacological sAC inhibition on ISO/ICI-induced hypertrophy of isolated cardiomyocytes. (A–D) Cardiomyocytes were cultured for 24 h under basal conditions or with the addition of ISO/ICI and/or KH7 (12.5 μmol/L). In (A) the cross sectional areas of 118 cells are given (n = 5). In (B) the RNA (ng/μL)/DNA (ng/μL) ratio (n = 3) and in (C) the protein (μg/μL)/DNA (ng/μL) ratio are presented (n = 3). (D) Western blot analysis followed by optical band density analysis of α-skeletal actin (n = 5). Data were normalized to GAPDH band density and to the control (no treatment, 100%). (A-D) Data are expressed as means ± SEM. *(P < 0.05) or ***(P < 0.001) vs. ISO/ICI-treated cells. (E) Isolated rat cardiomyocytes were incubated with ISO/ICI and KH7 (0.0, 6.0, 9.0, 12.5, 15.0 μmol/L) for 24 h. The protein (μg/μL)/ DNA (ng/μL) ratio was determined (n = 4 cardiomyocyte preparations). Data are expressed as means ± SEM. 6.0 and 9.0 μmol/L KH7 vs 0.0 μmol/L KH7: P<0.05; 12.5 and 15 μmol/L KH7 vs 0.0mKH7: P<0.01. n refers to the number of cardiomyocyte preparations.

As a first approach, we used ventricular myocytes isolated from the adult rat heart to reveal a possible function of sAC in cardiac ISO/ICI-induced hypertrophy. Isoprenaline activates β-adrenoceptors in general, i.e. β_1_- and β_2_-adrenoceptors. To exclude β_2_-adrenoceptor signaling, β_2_-adrenoceptors were blocked by ICI118,551. Upon stimulation of β_1_-adrenoceptors for 24 h hypertrophy of cardiomyocytes was clearly visible (Fig A in S1 Fig). Hypertrophic growth of cardiomyocytes was quantified by analysis of the cross sectional area of cells, and the RNA/DNA and protein/DNA ratios (Fig 1A–1C). All of the parameters were significantly elevated under ISO/ICI treatment for 24 h. The cross sectional area of cardiomyocytes was already slightly increased after 6 and 9 h, a strong significance was obtained after a 12 and 24 h treatment with ISO/ICI (Fig B in S1 Fig). Fittingly, expression of α-skeletal muscle actin, a biochemical marker of cardiac hypertrophy, was markedly enhanced under ISO/ICI-treatment after 24 h (Fig 1D). The hypertrophic response was accompanied by a significant elevation of intracellular Ca^2+^ concentration commonly known in cardiac hypertrophy (Fig C S1 Fig)

Inhibition of sAC activity with KH7 (12.5 μmol/L) prevented the ISO/ICI-induced hypertrophy, whereas KH7 alone exhibited no effect on the hypertrophic parameters investigated in control cells (Fig 1). Suppression of hypertrophic growth was dependent on the KH7 concentration (Fig 1E). Maximal effect on the protein/DNA ratio was obtained from 12.5 μM KH7 up. At 12.5μM KH7 all hypertrophic parameters investigated were near control values indicating a markedly suppression of hypertrophic growth (Fig 1A–1D). and apparently did not affect cell survival whereas it significantly reduced the total intracellular cAMP concentration in control cells by 50–60% (Fig A in S2 Fig). To examine whether ISO/ICI treatment for 24 h affected cellular cAMP, total cellular cAMP concentration was determined at the end of treatment. The analysis revealed no significant alteration of the cAMP concentration, which is due to strong PDE protection. Indeed incubation in presence of IBMX (500μmol/L), a pan-inhibitor of PDE, uncovered the effect of ISO/ICI treatment (Fig B in S2 Fig).

**Fig 2:**
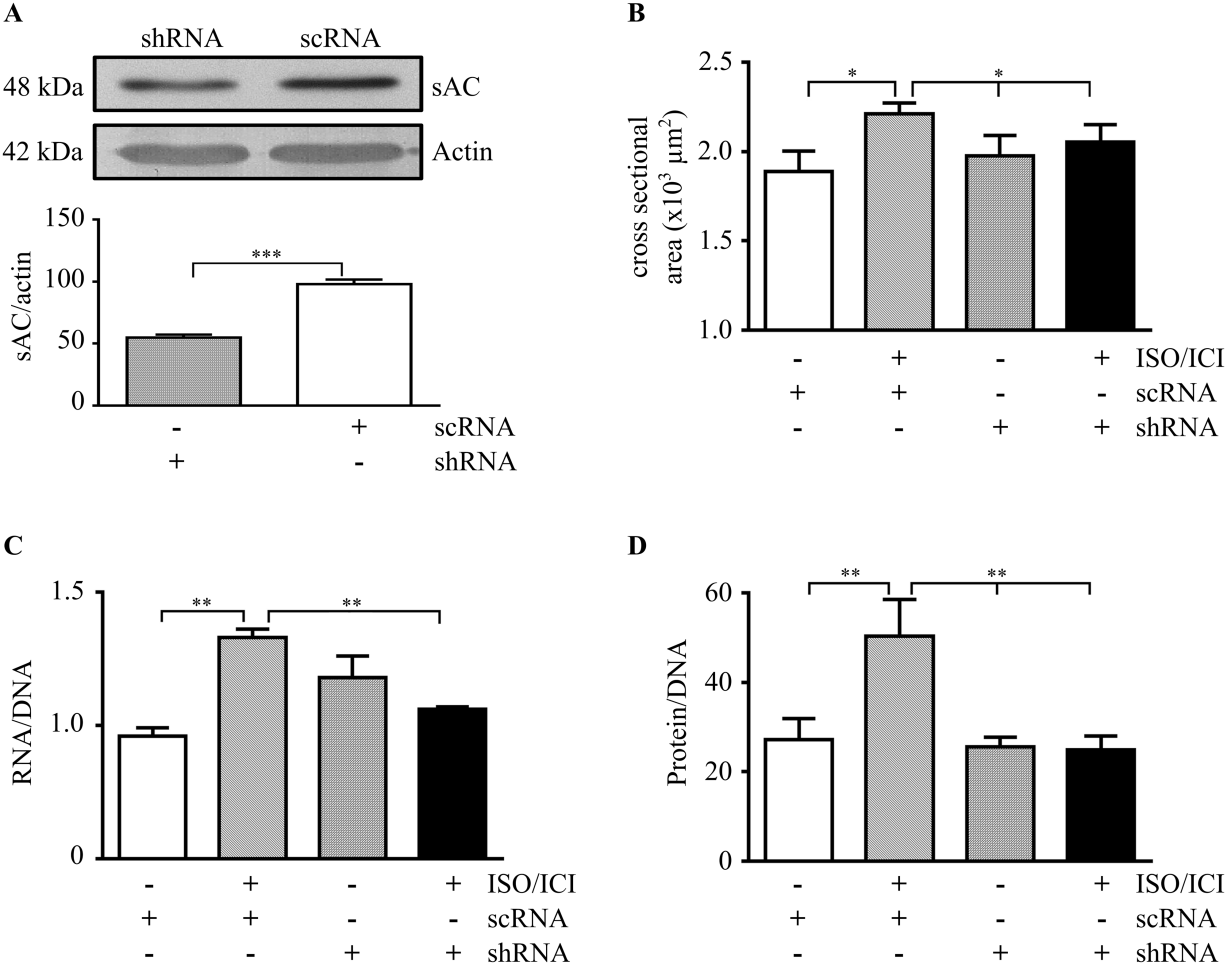
sAC knockdown in ISO/ICI treated isolated adult cardiomyocytes. (A) Representative western blots followed by the optical band analysis of sAC in lysates of cardiomyocytes after the following treatments: ISO/ICI (24h), adenoviral transfections either with scrambled shRNA (scRNA) or with sAC-targeted shRNA (shRNA). Data were normalized to actin band intensity. n = 5,*** (P<0.0001). (B-D) Statistical analyses of the cross sectional cell area of 110 cells from three cardiomyocyte preparations, the RNA/DNA ratio (n = 3) and the protein/DNA ratio (n = 3) are shown. *(P<0.05) and **(P<0.01),vs. ISO/ICI-treated cells expressing scRNA. *All* data are expressed as means ± SEM. n refers to the number of cardiomyocyte preparations analyzed.

To exclude that the attenuation of hypertrophic growth under sAC inhibition might be partly due to KH7 side effects or altered sAC expression, first, sAC expression was analyzed by western blot. sAC expression was not affected significantly (S3 Fig). Second, instead of inhibiting sAC activity by KH7, sAC expression was suppressed in isolated cardiomyocytes by adenovirus-mediated transfection of sAC-targeted shRNA. The transfection efficiency was > 80% (S4 Fig). Western blot analysis revealed a decrease in sAC protein levels by about 50% (Fig 2A). Similar to pharmacological suppression of sAC activity, the hypertrophic response, which was clearly observed in control cells (scrambled shRNA transfection), was nearly abolished by sAC knockdown (Fig 2B–2D).

**Fig 3:**
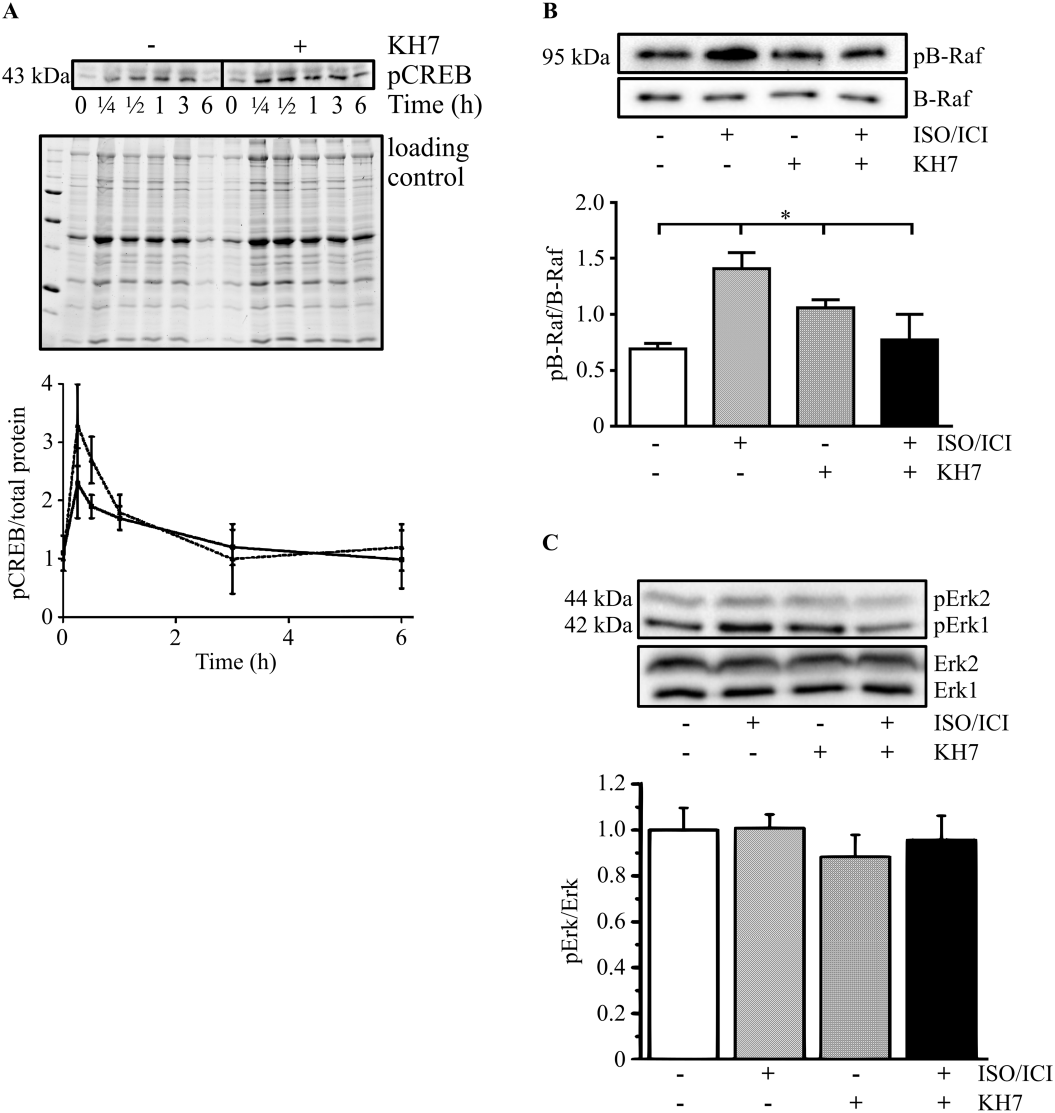
CREB, B-Raf, Erk1/2 phosphorylation in adult rat cardiomyocytes. (A) Western blot analysis of phosphorylated CREB (pCREB,) from the lysates of untreated (-KH7) or KH7-treated (+KH7, 12.5 μmol/L) cardiomyocytes followed by optical band density analysis at different incubation times with ISO/ICI (n = 4). Data were normalized to the total proteins by gel staining as indicated in Methods; incubation times: 0, 15 min, 30 min, 1 h, 3 h, 6 h. Solid line indicates control, broken line—treatment with KH7. Western blot analysis of (B) phosphorylated B-Raf (pB-Raf; Ser445) and B-Raf (n = 3), of (C) phosphorylated Erk1/2 (pErk1, pErk2; TEY motif)) and Erk1/2 (n = 4) followed by the optical band density analysis (lower panel) are presented. Data were normalized to total protein and are expressed as means ± SEM. *(P<0.05), vs. ISO/ICI treated control cells. n refers to the number of cardiomyocyte preparations analyzed.

To understand which underlying cellular mechanism may be involved in sAC-dependent hypertrophy, we examined the potential role of CREB as a downstream target of sAC. The addition of ISO/ICI led to a similar temporary rise of CREB phosphorylation within 30 min in control and KH7-treated cardiomyocytes (Fig 3A). Therefore, in our model, ISO-induced CREB phosphorylation appears to be sAC-independent.

Since our recent reports demonstrated that B-Raf is an important downstream target of sAC [19,23], we investigated whether phosphorylation of B-Raf was also affected by sAC inhibition in our model. We found that phosphorylated B-Raf was markedly enhanced upon long-term treatment., i.e. for 24 h with ISO/ICI. This activating effect was abolished by inhibition of sAC with KH7 (Fig 3B).

Surprisingly, phosphorylation of Erk1/2 (TEY), a downstream target of B-Raf was not altered under ISO/ICI treatment (Fig 3C), indicating that ISO/ICI induced hypertrophy is probably not promoted via Erk.

### KO of sAC prevented pressure overload-induced hypertrophy in mice

To strengthen the findings obtained with an *in vitro* model, we applied TAC, a widely used *in vivo* model of cardiac hypertrophy, for 2 weeks, to generate chronic pressure overload in WT or sAC KO mice. Western blot analysis of sAC expression confirmed its presence in the heart of WT mice and its absence in KO mice (supporting information S5 Fig). TAC led to nearly equal mortality rates in KO (27.6%) and WT mice (21.7%).

**Fig 4:**
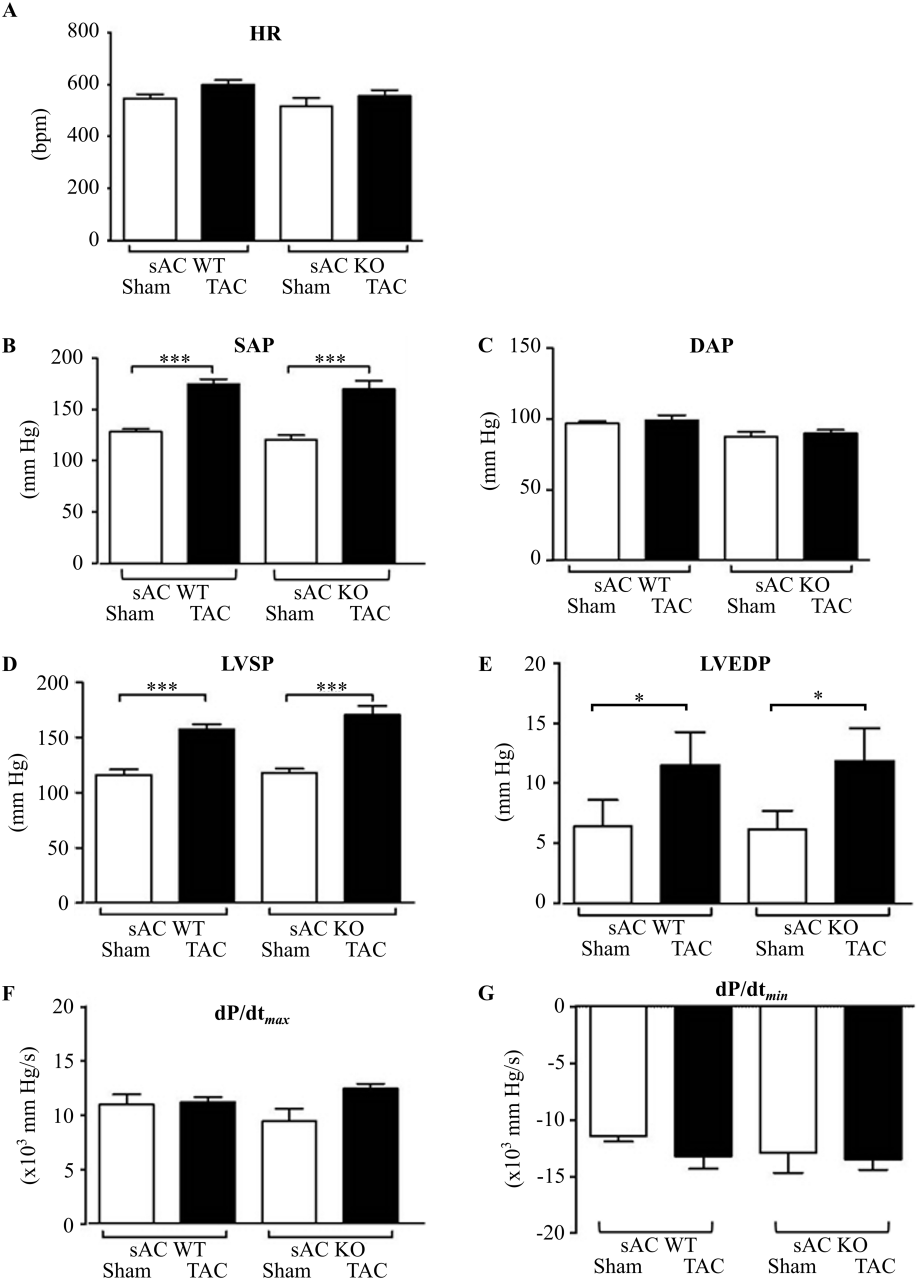
Effect of TAC on haemodynamic parameters in WT and sAC-KO mice. Analyses of (A) heart rate (HR), (B) systolic (SAP), (C) diastolic aortic pressure (DAP), (D) left ventricular systolic pressure (LVSP), (E) left ventricular end-diastolic pressure (LVEDP), rates of (F) contraction (+dp/dt) and (G) of relaxation (–dp/dt) were performed in anaesthetised WT or sAC KO mice 2 weeks after TAC or sham surgery. Data are expressed as means ± SEM, n = 9–12, ***(P< 0.001), *(P<0.05).

Analysis of haemodynamic parameters revealed no significant differences between WT or KO mice before and 2 weeks after TAC (Fig 4).

**Fig 5:**
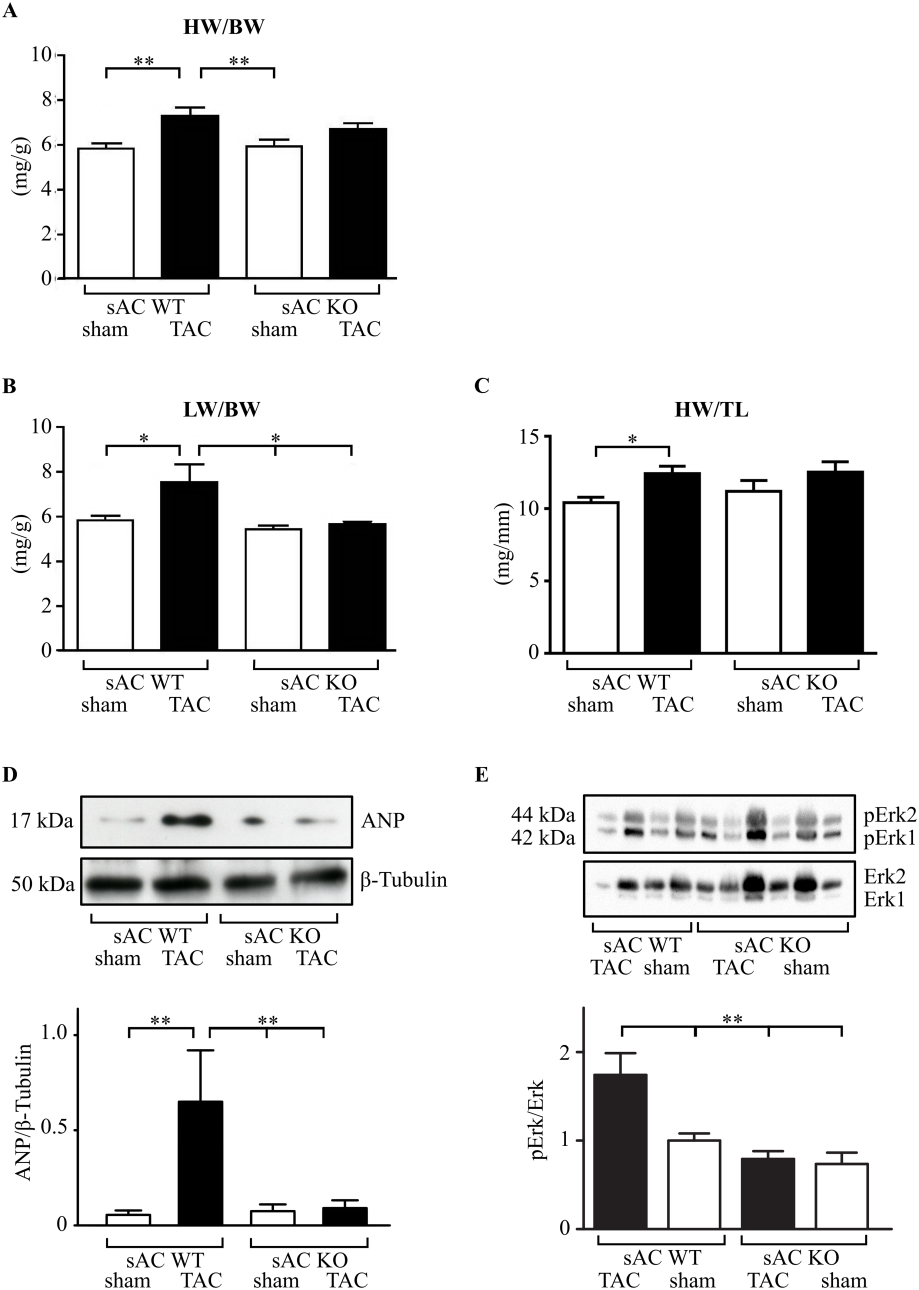
Hypertrophic parameters in sham and TAC operated WT and sAC KO mice. (A-C) Body weight, lung weight, heart weight and Tibia length (BW, LW, HW, TL, respectively) and their ratios (HW/BW;LW/BW and HW/TL) were analyzed 2 weeks after TAC or sham surgery in WT or sAC KO mice (n = 9–12). *(P< 0.05); **(P < 0.01). (D) Western blot analysis of ANP followed by optical band density analysis (n = 3 for sAC-WT and n = 5 for sAC-KO), ANP is normalized to tubulin. Equal experimental conditions as in A-C. Data are expressed as means ± SEM. **(P < 0.01) vs. TAC in wt mice. (E) Western blot analysis of pERK1/2 followed by optical band density analysis performed with cardiac tissue lysates from WT sham (n = 4), sAC WT TAC (n = 2), sAC KO sham (n = 4) and sAC KO TAC (n = 5) mice. Differences in total Erk1/2 band intensities are due to different total protein concentrations. Data are expressed as means ±SEM; **(P < 0.01) vs. TAC in WT mice.

TAC significantly increased the heart weight to body weight ratio, the lung weight to body weight and heart weight to tibia length ratios in WT mice, but not in KO mice (Fig 5A, 5B and 5C). Body weights of mice investigated were not significantly different in all groups (see methods) as are the thickness of ventricular wall (S6 Fig). Additionally, TAC led to the expression of ANP, a fetal cardiac hormone and molecular marker for pathological hypertrophy, which was prevented by sAC KO (Fig 5D).

Mechanistic analysis revealed a significant elevation of phosphorylated Erk1/2 (TEY motif) only in TAC WT mice, but not in KO mice (Fig 5E). These results imply that TAC-induced Erk1/2 phosphorylation is sAC dependent.

## Discussion

In this study we applied two different approaches to elucidate the role of sAC in cardiac hypertrophy. On the one hand, we investigated the effects of either pharmacological inhibition of sAC or sAC knockdown in isolated adult rat cardiomyocytes treated with ISO/ICI (*in vitro* model). On the other hand, we examined sAC KO mice that underwent TAC compared to WT mice (*in vivo* pressure overload model).

To investigate the role of sAC in cardiomyocytes we used the sAC-specific inhibitor KH7. Though KH7 does not affect tmACs, guanylyl cyclase, and phosphodiesterases (PDE) up to concentrations of 100 μmol/L [29], possible side effects of KH7 in cells have not been described so far.

In ISO/ICI-treated cardiomyocytes, submaximal inhibition of sAC with KH7 (12.5 μmol/L) abolished the hypertrophic response. This effect of sAC inhibition is not due to negative effects of KH7 on cell viability. Furthermore, KH7 had no effect on non-hypertrophic control cells and a nearly identical response was obtained by suppressing sAC expression using sAC-specific shRNA. In addition, sAC expression in cardiomyocytes remained nearly identical under all experimental conditions applied. Thus, the current study suggests a novel mechanism involved in cardiac hypertrophy, i.e., a sAC-generated cAMP pool.

cAMP signalling has been shown for several cardiac pathologies including cardiac hypertrophy [33]. Nevertheless, the data are controversial, and a pro-hypertrophic as well as an anti-hypertrophic role of cAMP-signaling has been shown [34–36]. This discrepancy may be due to differences in models or treatments.

Additionally, cAMP signalling demonstrates a highly organized intracellular compartmentalization of cAMP producing (tmAC and sAC) and degrading (PDE) enzymes as well as main downstream effectors (PKA and Epac) [23,37], and the net effect of cAMP signaling activation is dependent on elevation of cAMP in particular microdomains.

Indeed, the recent report of Zoccarato et al. [36], demonstrates that elevation of cAMP by inhibition of PDE2 suppresses cardiac hypertrophy *in vitro* (ß-adrenergic stimulation) and *in vivo* (TAC), whereas inhibition of PDE3 and PDE4 promotes hypertrophic growth. Although the authors did not define the source(s) for cAMP, the current study implies that sAC-generated cAMP might be involved in the pro-hypertrophic response.

In agreement with the compartmentalization hypothesis, 24 h treatment with ISO/ICI in our study significantly elevated total cellular cAMP concentration only in the presence of the pan-inhibitor of PDEs, IBMX. Thus, a strong PDE protection is present in cardiomyocytes, which is an essential requirement for building distinct cAMP compartments. Though we were not able to detect cAMP elevation under ISO/ICI treatment, we cannot exclude that in certain subcellular compartments cAMP may be elevated, which however remains undetectable with cAMP analysis in the whole cell lysate.

Next, we attempted to elucidate the underlying cellular mechanism involved in sAC dependent hypertrophic growth. According to Zippin et al. [25], CREB is an important target of nuclear sAC, at least in tissues other than heart muscle. In the heart, CREB phosphorylation and activation occurs as an early response to a hypertrophy stimulus [26]. Harrison et al. [38] showed CREB activation after about 20 min upon addition of endothelin-1 to isolated neonatal cardiomyocytes to induce hypertrophy, which is in accordance with our findings. We observed peak CREB phosphorylation about 20–30 min after addition of ISO/ICI. However, no significant changes in ISO/ICI-induced CREB phosphorylation occurred in the presence of the sAC inhibitor KH7, indicating that sAC might only be marginally responsible for CREB phosphorylation in ISO/ICI-treated cardiomyocytes. Indeed, CREB can be phosphorylated by several protein kinases, such as CaMKII [39] and MAPK [40], both of which play a major role in pathological hypertrophy [41,42]. Furthermore, CaMKII is activated by elevated cytosolic Ca^2+^ concentration [43]. We observed a significantly increased intracellular Ca^2+^ concentration upon long-term treatment with ISO/ICI (Fig C in S1 Fig). This may be due to IP_3_ or rhyanodine receptor phosphorylation by PKA leading to a leaky receptor. Ca^2+^ is also known as an important signal for hypertrophy [44]. Furthermore elevated Ca^2+^ may lead to activation of sAC, as has been shown previously in other cell types [6–9,45].

In addition to PKA, Epac is another important target downstream of sAC. Recently, we found that sAC via Epac/Rap1 and its direct target B-Raf promotes the proliferation of tumour and non-tumour epithelial cells [19,24]. According to Vidal et al. [1], the Raf cascade is activated by G_sα_ and G_βγ_ subunits in ISO-induced hypertrophy in neonatal cardiomyocytes of mice. Gelb and Tartaglia [16] summarize that Ras/MAPK pathways activated by either Raf1 or B-Raf are involved in cardiac hypertrophy. In accordance with these data, we found that phosphorylation of B-Raf was significantly increased upon treatment of rat cardiomyocytes with ISO/ICI. Similarly to findings in prostate cancer and epithelial cells [19,24], inhibition of sAC activity by KH7 reduced B-Raf phosphorylation in ISO/ICI-treated cardiomyocytes. Thus in our model phosphorylation of B-Raf is regulated by sAC in adult rat cardiomyocytes, and might be involved in the sAC-dependent hypertrophy under chronic ISO/ICI stimulation. Surprisingly, phosphorylated Erk, the only known target of B-Raf, was not altered in adult rat cardiomyocytes under chronic ISO/ICI stimulation. These findings are in contrast to Vidal et al [1], who described Erk-promoted hypertrophic growth upon chronic ISO stimulation. However, they used neonatal cardiomyocytes in contrast to adult cardiomyocytes applied in the present study. Our results suggest the involvement of other pathways in controlling cardiomyocyte hypertrophy under ISO/ICI treatment in adult cardiomyocytes. For example sAC might be involved in Ca^2+^ dependent pathways to induce hypertrophy. Such pathways might include CaMKII and/or calcineurin, which may be regulated via Epac/Rap2 [46]. Another possibility in promoting ISO/ICI induced hypertrophy is by inhibiting glycogen synthase kinase upon PKA phosphorylation [47].

Although chronic stimulation of β-adrenoceptors leads to diseases accompanied by cardiac hypertrophy, hypertension is the major cause of hypertrophy in the clinical setting. It has been shown that cAMP signalling plays an important role also in hypertension-induced hypertrophy [34,48]. Therefore, to further substantiate the role of sAC in cardiac hypertrophy, we applied TAC-induced pressure overload in mice. The sAC KO mice showed no obvious haemodynamic abnormalities, and pressure overload was equally developed in both mouse types. In contrast, WT mice exclusively developed a hypertrophic phenotype, i.e., elevation of HW/BW and HW/TL ratios. Surprisingly, no effect of TAC on the wall thickness in WT mice was found. We suppose that the moderate TAC model applied in our study, i.e., elevation of HW/BW by 30% in WT mice, was not sufficient to detect an alteration in the wall thickness. In agreement, a lower sensitivity of the wall thickness (elevation by 22–25%) compared to the HW/BW (elevation by 80%) has been previously reported [49]. Similarly, a moderate level of the HW/TL elevation without significant alteration of the wall thickness has also been observed by others [50]. Furthermore, since the wall thickness is increasing with time after TAC [51], we suggest that 2 weeks of TAC in our study was not sufficient for a significant elevation of this particular hypertrophy marker. In contrast, expression of ANP, a marker of hypertrophy at the initial phase [52], was clearly enhanced in WT, but not in KO mice that underwent TAC.

Mechanistic analysis revealed that Erk phosphorylation was significantly enhanced in TAC WT mice, but not in the sAC KO mice, suggesting that Erk is involved in the sAC induced hypertrophy in the *in vivo* model. The importance of Erk for the hypertrophic response is underlined by a study of Ulm et al. [53], who showed that in Erk2 knock out mice hypertrophy was suppressed after 2 weeks of TAC.

Although probably different pathways in sAC induced hypertrophy are involved in the *in vitro* (ISO/ICI-treated) and *in vivo* (TAC-treated) models, the suppression of sAC inhibited the hypertrophic response to stress in both models.

In summary, we revealed a novel role of sAC in cardiac hypertrophy induced by β-adrenergic stimulation or by pressure overload. Erk might be a downstream target of sAC-dependent hypertrophy in TAC treated mice.

## Supporting information

S1 Fig Effect of ISO/ICI treatment on cardiomyocytes (A), hypertrophy dependent on incubation time (B) and intracellular Ca^2+^ (C). (PDF)

S2 Fig Effect of KH7 on cellular cAMP (A, B). (PDF)

S3 Fig sAC expression in adult rat cardiomyocytes. (PDF)

S4 Fig Transfection of isolated adult rat cardiomyocytes. (PDF)

S5 Fig Expression of sAC in wild type and sAC-knockout mice hearts. (PDF)

S6 Fig Wall thickness of male WT (sham/TAC) and sAC- KO (sham/TAC) mice. (PDF)

S1 Data Data tables underlying the figures based on *in vitro* experiments. Supporting data underlying Figs 1–3 of the main manuscript and S1–S3 Figs of supporting informations are given.
(PDF)

S2 Data Data tables underlying the figures based on *in vivo* experiments. Excel table 1 contains supporting informations underlying Figs 4 and 5 in the main manuscript including statistical power calculation, excel table 2 shows data underlying supporting figure S6 Fig.
(PDF)

## Abbreviations

(p)B-Raf: (phosphorylated) rapidly activated fibrosarcoma
(p)CREB: (phosphorylated) cAMP response element binding protein
(p)Erk: (phosphorylated) extracellular-signal regulated kinase
ANP: atrial natriuretic peptide
AR: adrenoceptor
B(H, L)W: body (heart, lung) weight
DAP: diastolic arterial pressure
Epac: exchange protein activated by cAMP
HR: heart rate
ISO: isoprenaline
KO: knockout
LVEDP: left ventricular end-diastolic pressure
LVSP: left ventricular systolic pressure
PDE: phosphodiesterase
r.u.: relative units
sAC: soluble adenylyl cyclase
SAP: systolic aortic pressure
scRNA: scrambled shRNA
shRNA: small hairpin RNA
sk: skeletal
TAC: transverse aortic constriction
tmAC: membrane bound adenylyl cyclase
WT: wild type
