# Supplementary material for: Soluble adenylyl cyclase: A novel player in cardiac hypertrophy induced by isoprenaline or pressure overload
Source: PLoS One. 2018 Feb 21;13(2):e0192322. doi: 10.1371/journal.pone.0192322 (PMC5821345; doi:10.1371/journal.pone.0192322)

S1 Fig

Effect of ISO/ICI treatment on cardiomyocytes (A), hypertrophy dependent on incubation time (B) and intracellular Ca^{2+} (C)

(A) Representative light microscopic images (objective 40×) of single isolated adult rat cardiomyocytes after 24 h in culture, untreated (Ctl), or treated with 10 µmol/L ISO (isoprenaline) and 0.05 µmol/L ICI 118,551 (ICI). **(B)** Freshly isolated adult rat cardiomyocytes (100 000 cells/dish and time point) were incubated with ISO (10µmol/L)/ICI 118,551 (0.05µmol/L). Directly after drug addition (0 h), then after 3, 6, 12 and 24 h cells size was measured. Data are means ± SEM from 3 preparations, 150 cells measured in total. ** P<0.01 vs. 0 h. After 12 and 24 h a significant difference in growth was obtained. **(C)** Intracellular Ca^{2+} concentration (Indo-1, presented as an emission ratio at 475 nm (F) to 400 nm (F0), relative units) analysed in cardiomyocytes 24 h after incubation with ISO/ICI. Data are expressed as means ± SEM; *** P < 0.001 (one way ANOVA; n=5).

A

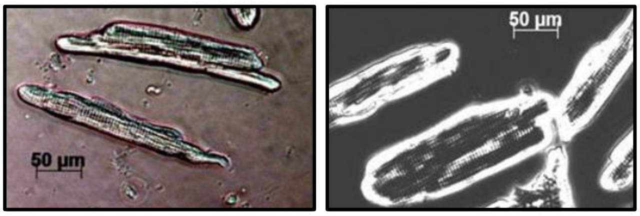

Ctl ISO/ICI

B

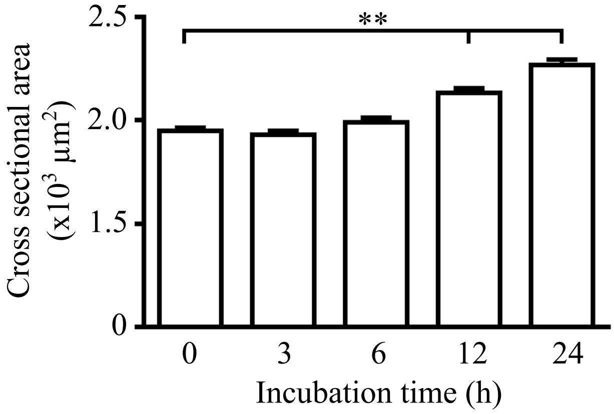

C

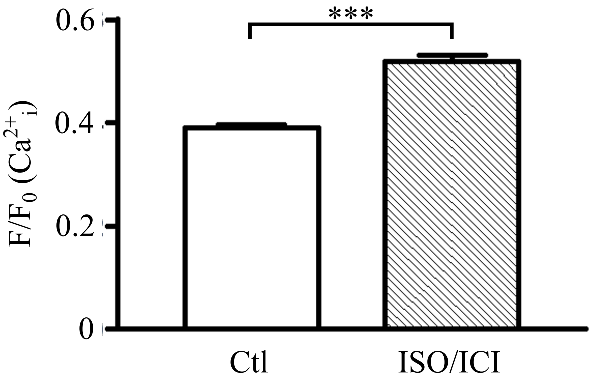

Supplement: S1 Fig — (PDF) [file pone.0192322.s001.pdf]
