# Supplementary material for: Soluble adenylyl cyclase: A novel player in cardiac hypertrophy induced by isoprenaline or pressure overload
Source: PLoS One. 2018 Feb 21;13(2):e0192322. doi: 10.1371/journal.pone.0192322 (PMC5821345; doi:10.1371/journal.pone.0192322)

S2 Fig

Effect of KH7 on cellular cAMP in isolated rat cardiomyocytes

cAMP was determined using the cAMP-EIA kit (Cayman Chemicals, MI, USA) according to the manufacturer's instructions. 500 000 cardiomyocytes from freshly isolated adult rat ventricles were incubated **(A)** with KH7 (0, 5, 12.5, 15, 20 and 40 µmol/L each) prior to cAMP determination or **(B)** with or without ISO/ICI or the general phosphodiesterase inhibitor IBMX (500 µmol/L). Data are presented as means ± SEM, *** (P<0.001), n.s. (P>0.05).

A

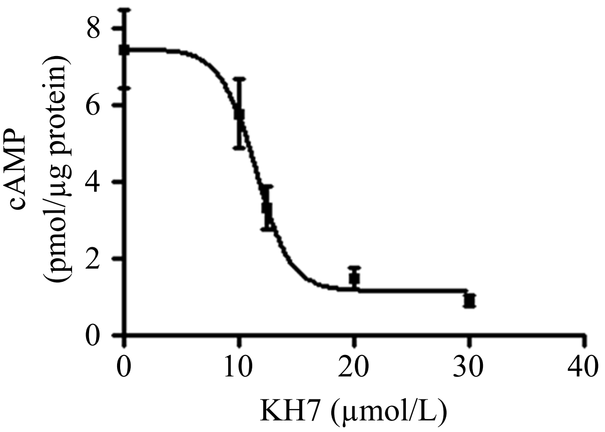

B

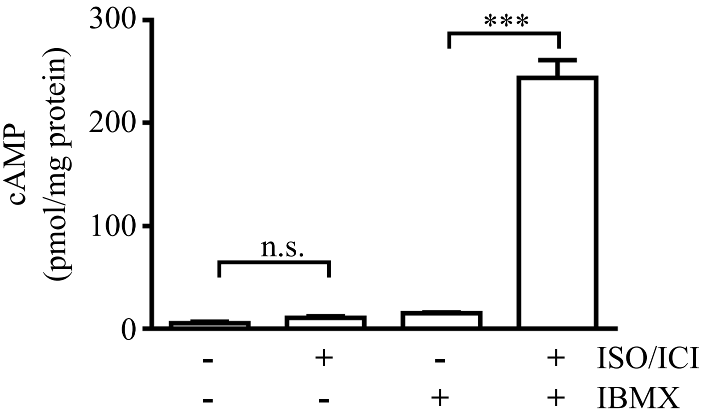

Supplement: S2 Fig — (PDF) [file pone.0192322.s002.pdf]
