# Supplementary material for: Soluble adenylyl cyclase: A novel player in cardiac hypertrophy induced by isoprenaline or pressure overload
Source: PLoS One. 2018 Feb 21;13(2):e0192322. doi: 10.1371/journal.pone.0192322 (PMC5821345; doi:10.1371/journal.pone.0192322)

S3 Fig

sAC expression in adult rat cardiomyocytes

Representative western blots followed by the optical band analysis of sAC in lysates of control, i.e. untreated cardiomyocytes or after treatment with ISO/ICI (24 h), KH7 (24 h), ISO/ICI+KH7 (24 h). n = 5. n.s. (P>0.05) vs. control. Data are expressed as means ± SEM. n refers to the number of cardiomyocyte preparations analysed.

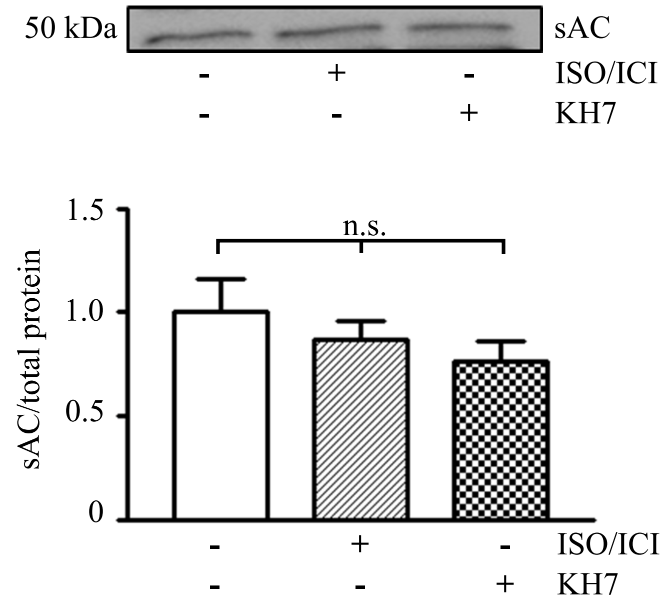

Supplement: S3 Fig — (PDF) [file pone.0192322.s003.pdf]
