# Supplementary material for: Soluble adenylyl cyclase: A novel player in cardiac hypertrophy induced by isoprenaline or pressure overload
Source: PLoS One. 2018 Feb 21;13(2):e0192322. doi: 10.1371/journal.pone.0192322 (PMC5821345; doi:10.1371/journal.pone.0192322)
Supplement: S4 Fig — (PDF) [file pone.0192322.s004.pdf]

S4 Fig

Transfection of isolated adult rat cardiomyocytes

Representative light microscopy images (LM, objective left: 40×; right: 10×) and fluorescence images of enhanced green fluorescent protein (eGFP) in cardiomyocytes 4 days after treatment with 100 µl of a recombinant adenovirus solution to co-express sAC specific small hairpin RNA and eGFP. The expression efficiency of scrambled RNA was nearly identical.

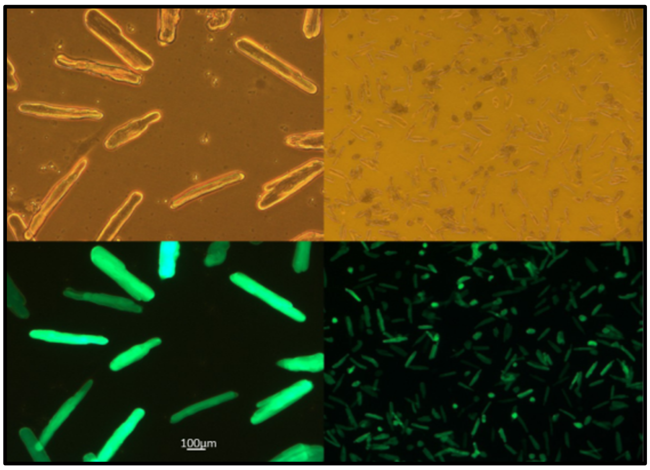

LM

eGFP fluorescence (em 509 nm)

40x 10x
