# Supplementary material for: Soluble adenylyl cyclase: A novel player in cardiac hypertrophy induced by isoprenaline or pressure overload
Source: PLoS One. 2018 Feb 21;13(2):e0192322. doi: 10.1371/journal.pone.0192322 (PMC5821345; doi:10.1371/journal.pone.0192322)

S5 Fig

Expression of sAC in wild type and sAC-knockout mice

Representative western blot analysis of sAC. Western blots were performed from the lysates of hearts from wild type (WT) and sAC knockout (KO) mice. The sAC specific band (50 kDa) is indicated by an arrow.

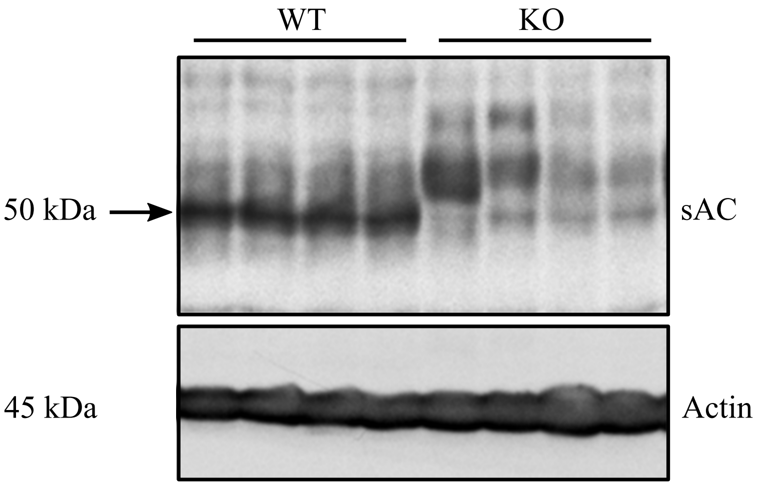

Supplement: S5 Fig — (PDF) [file pone.0192322.s005.pdf]
