# Supplementary material for: Soluble adenylyl cyclase: A novel player in cardiac hypertrophy induced by isoprenaline or pressure overload
Source: PLoS One. 2018 Feb 21;13(2):e0192322. doi: 10.1371/journal.pone.0192322 (PMC5821345; doi:10.1371/journal.pone.0192322)

S6 Fig

Wall thickness of male WT- (Sham/TAC) and sAC-KO (sham/TAC) mice

In **(A)** an image of a 5 µm slice, stained with Masson's trichrome, is shown. The arrows and numbers designate the region of measurement. (**B**). Means ± SEM of wall thickness (1+2) in mm are given for WT+sham (n=4), WT+TAC (n=6) and sAC-KO +sham/TAC (n=5 each). Data were analyzed using two way ANOVA. Differences in values between the groups were not significant.

A

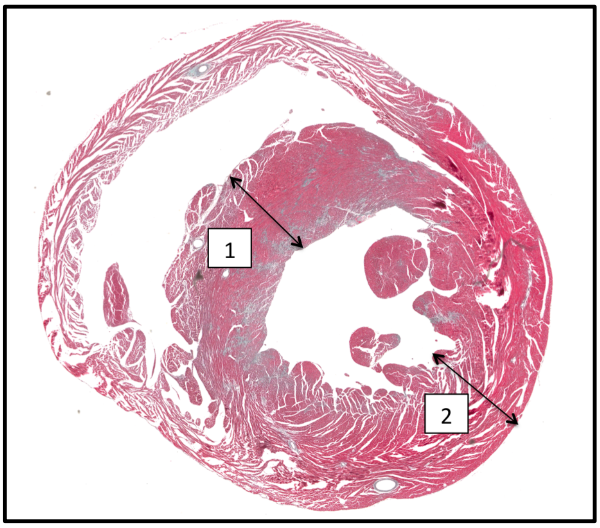

B

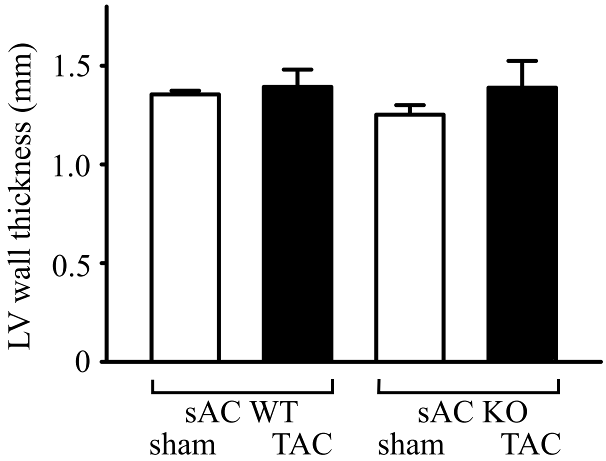

Supplement: S6 Fig — (PDF) [file pone.0192322.s006.pdf]
