# Supplementary material for: Soluble adenylyl cyclase: A novel player in cardiac hypertrophy induced by isoprenaline or pressure overload
Source: PLoS One. 2018 Feb 21;13(2):e0192322. doi: 10.1371/journal.pone.0192322 (PMC5821345; doi:10.1371/journal.pone.0192322)
Supplement: S1 Data — Supporting data underlying Figs 1–3 of the main manuscript and S1–S3 Figs of supporting informations are given. (PDF) [file pone.0192322.s007.pdf]

Data Table underlying Fig 1:

Effect of pharmacological sAC inhibition on ISO/ICI-induced hypertrophy of isolated cardiomyocytes.

Data were obtained as described in method section of the manuscript.

**Fig. 1A Mean cross sectional area of adult cardiomyocytes (μm^2) ± SEM; (n=118 (5 preparations))**				**statistics**
Control (column A)	*+ISO/ICI (column B)*	*+KH7 (12.5µM) (column C)*	*+ISO/ICI+KH7 (column D)*	One way Anova with Holm-Sidak post test
2.07 ± 0.02	2.36 ± 0.02	2.16 ± 0.02	2.22 ± 0.02	B vs A ***P< 0.0001; B vs C ***P< 0.0001; B vs D ***P< 0.0001
**Fig. 1B Mean ratio RNA/DNA ± SEM (n=3)**				One Way ANOVA
0.98 ± 0.062	2.89 ± 0.46	0.83 ± 0.03	0.74 ± 0.03	***P<0.0001
**Fig. 1C Mean ratio Protein/DNA ± SEM (n=3)**				One Way ANOVA
19.08 ± 0.99	34.91 ± 3.28	19.29 ± 2.07	18.71 ± 2.26	***P= 0.0002
**Fig. 1D Relative α-skeletal actin expression (%) ± SEM;** (Analysis of western blots with cardiomyocyte lysates from n=5 cardiomyocyte preparations); data were normalized to GADH band intensities (loading control) and control cells (=100 %)				One Way ANOVA
100 ± 2.02	150.57 ± 8.88	102.85 ±16.86	102.66 ± 13.64	*P=0.0209

**Fig. 1E Mean ratio Protein/DNA ± SEM (n= 4)**<br>**KH7 concentration (µmol/L)**					One Way Anova<br>**P=0.006
0 (control)	6.0	9.0	12.5	24.0	control vs each other KH7 concentration:
3.60 ± 0.32	2.16 ± 0.63	2.15 ± 0.41	1.75 ± 0.41	1.73 ± 0.18	6.0 and 9.0 µmol/L KH7: *P<0.05<br>12.5 and 15 µmol/L KH7: **P<0.01

Data tables underlying Fig 2:

sAC knockdown in ISO/ICI treated isolated adult cardiomyocytes.

**Fig. 2A Relative sAC expression (%) ± SEM after sAC specific sh-RNA and scrambled RNA (sc-RNA) transfection**<br>(Analysis of western blots with cardiomyocyte lysates from n=5 cardiomyocyte preparations); data were normalized to actin band intensities (loading control) and sc-RNA (=100 %)				**Statistics**
sh-RNA<br>55.60 ± 12.23	sc-RNA<br>100 ± 13.43			t-Test (unpaired)<br>***P<0.0001
**Fig. 2B Mean cross sectional area of adult cardiomyocytes (µm²) ± SEM; (n=110 (3 cardiomyocyte isolations))**				One way Anova : *P=0.0495
Control (scRNA) (column A)	*+ISO/ICI (column B)*	*shRNA (column C)*	*shRNA/ISO/ICI (column D)*	t-Test (unpaired)
1.89 ± 0.11	2.21 ±0.06	1.98 ± 0.098	2.05 ±0.05	B vs D *P= 0.043; B vs A *P= 0.012; B vs C *P= 0.041
**Fig. 2C Mean ratio RNA/DNA ± SEM (n=3)**				One Way ANOVA
0.96 ± 0.03	1.33 ± 0.03	1.18 ± 0.08	1.06 ± 0.01	**P=0.0023; A, D vs B: **P<0.01; B vs C not significant
**(D) Mean ratio Protein/DNA ± SEM (n=3)**				One Way ANOVA<br>*** P= 0.0002
27.23 ± 4.71	50.36 ± 8.19	25.61 ± 2.13	24.95 ± 3.04	B vs A,C,D **P<0.01

Data tables underlying Fig 3:

CREB, B-Raf, Erk1/2 phosphorylation in adult rat cardiomyocytes.

**Fig. 3A Relative Creb phosphorylation ± SEM normalized to total protein (loading control) dependent on incubation times with ISO/ICI + or – KH7 (12.5 µmol/L)**							**Statistics**
Incubation times (h)							t-Test (unpaired)
0	1/4	1/2	1	3	6		Comparison of each time point (A) vs (B)
1.1 ± 0.3 (n=4)	2.3 ± 0.6 (n=4)	1.9 ± 0.2 (n=2)	1.7 ± 0.2 (n=4)	1.2 ± 0.3 (n=2)	1.0 ± 0.5 (n=4)	*+KH7 (A)*	Not significantly different P>> 0.05
1.0 ± 0.01 (n=4)	3.3 ± 0.7 (n=4)	2.7 ± 0.4 (n=2)	1.8 ± 0.3 (n=4)	1.0 ± 0.6 (n=2)	1.2 ± 0.4 (n=4)	*-KH7 (B)*	

**Fig 3B Relative B-Raf phosphorylation ± SEM normalized to total B-Raf (loading control) (n=3 cardiomyocyte preparations)**				One way Anova
Control (column A)	*+ISO/ICI (column B)*	*+KH7 (12.5µM) (column C)*	*+ISO/ICI+KH7 (column D)*	
0.69 ± 0.05	1.41 ± 0.14	1.06 ± 0.07	0.77 ± 0.23	*P= 0.026/ column B vs A,D: *P<0.05

**(C) Relative Erk1/2 phosphorylation (TEY motif) ± SEM normalized to total Erk1/2 (loading control) and control cells (=1) (n=6 cardiomyocyte preparations)**				One Way ANOVA
Control *(column A)*	***+ISO/ICI*** *(column B)*	*+KH7 (12.5µM) (column C)*	*+ISO/ICI+KH7 (column D)*	
1 ± 0.10	1.01 ± 0.06	0.88 ± 0.1	0.96 ± 0.11	P=0.76, no significant differences

Data Tables underlying supporting informations S1Fig B and C:

Effect of ISO/ICI treatment on cardiomyocytes (A), hypertrophy dependent on incubation time (B) and intracellular Ca^{2+} (C)

**(B) Mean Protein/DNA ratio ± SEM (n=3 cardiomyocyte preparations and in total 150 cells were sized)**					**statistics**
Incubation time with ISO/ICI (h)					One Way ANOVA P<0.0001 ** P<0.01: 12, 24h vs 0 h; 3 and 6 h not significant different vs 0 h
0	*3*	*6*	*12*	*24*	
1.95 ± 0.02	1.93 ± 0.02	1.99 ± 0.02	2.14 ± 0.02	2.64 ± 0.03	

**(C) Intracellular Ca^{2+} concentration (Indo-1), presented as a mean emission ratio 475 nm (F) to 400 nm (F_0), relative units ± SEM (n=5 preparations and 50 cells were analysed in total)**		
Control, after 24 h in culture, untreated (F/F_0)	*Cardiomyocytes treated with ISO/ICI for 24 h (F/F_0)*	Students T-test (unpaired) ***P<0.0001
0.39 ± 0.06	0.52 ± 0.01	

Data tables underlying supporting informations S2 Fig:

Effect of KH7 on cellular cAMP in isolated adult rat cardiomyocytes (A, B)

**(A) mean intracellular cAMP in cardiomyocytes treated with different concentrations of KH7 (µmol/L) ± SEM**					Nonlinear regression logEC50: 11.5µmol/L
Control, untreated 0	*10*	*12.5*	*20*	*30*	
2.67 ±0.03 (n=4)	2.14 ± 0.02 (n=2)	1.99 ± 0.02 (n=2)	1.93 ± 0.02 (n=2)	1.95 ± 0.02 (n=4)	

**(B) mean intracellular cAMP in control and ISO/ICI treated (24 h) cardiomyocytes with IBMX (500µmol/L) and without IBMX**				One way ANOVA
Control (column A)	*+ISO/ICI (Column B)*	*control + IBMX (Column C)*	*+ ISO/ICI +IBMX (column D)*	P>0.05: A vs B, C: not significant
5.17 ± 1.4 (n=4)	10.47 ± 1.4 (n=4)	14.77 ± 0.91 (n=5)	243.55 ±17.5 (n=5)	***P<0.0001 A,B,C vs D

Data Table underlying supporting informations S3 Fig:

sAC expression in adult rat cardiomyocytes

**SAC expression ± SEM normalized to total protein (loading control) and control cells (=1) analyzed by western blot (n=6)**			**Statistics**
Control, lysates of untreated cardiomyocytes	*Lysates of Cardiomyocytes treated with ISO/ICI for 24 h*	*Lysates of Cardiomyocytes treated with KH7 for 24 h*	One Way ANOVA<br>P= 0.4, differences are not significant
1.0 ± 0.4	0.9 ± 0.2	0.8 ± 0.2	
